# Retraction Note: Non-thermal plasma inhibits human cervical cancer HeLa cells invasiveness by suppressing the MAPK pathway and decreasing matrix metalloproteinase-9 expression

**DOI:** 10.1038/s41598-024-63655-y

**Published:** 2024-06-04

**Authors:** Wei Li, K. N. Yu, Lingzhi Bao, Jie Shen, Cheng Cheng, Wei Han

**Affiliations:** 1 Center of Medical Physics and Technology, Hefei Institutes of Physical Sciences, Anhui Province, Chinese Academy of Sciences, 350 Shushanhu Road, Hefei, 230031 P.R. China; 2 Institute of Plasma Physics, Hefei Institutes of Physical Sciences, Anhui Province, Chinese Academy of Sciences, 350 Shushanhu Road, Hefei, 230031 P.R. China; 3 Department of Physics and Materials Science, City University of Hong Kong, Tat Chee Avenue, Kowloon Tong, Hong Kong; 4 Collaborative Innovation Center of Radiation Medicine of Jiangsu Higher Education Institutions and School for Radiological and Interdisciplinary Sciences (RAD-X), Soochow University, Suzhou, Jiangsu China

Retraction of: *Scientific Reports* 10.1038/srep19720, published online 28 January 2016

The Editors have retracted this Article.

After the publication of this Article, concerns about overlapping image panels were brought to the attention of the Editors. Specifically:

- In Figure 5a, there is an area of partial overlap between the panels for Control and 10s.
- In Figure 8, there is an area of partial overlap between the panels for SCH772984 and SB203580.

The Editors no longer have confidence in the reliability of the data and the conclusions drawn.

Wei Li disagrees with this retraction. Wei Han did not explicitly state whether they agree or disagree with this retraction. The remaining authors did not respond to correspondence from the Publisher about this retraction.

